# Exploring the implementation of a data trust committee: a qualitative evaluation of processes and practices

**DOI:** 10.1186/s40900-025-00693-4

**Published:** 2025-03-06

**Authors:** Syka Iqbal, Sophie Moniz, Fiona Bennin, German Alarcon Garavito, Rosaline de Koning, Rosamund Yu, Cecilia Vindrola-Padros

**Affiliations:** 1 Rapid Research Evaluation and Appraisal Lab (RREAL), Department of Targeted Intervention, University College London, London, UK; 2 Department of Targeted Intervention, University College London, London, UK; 3 NIHR biomedical Research Centre, University College London Hospital (UCLH) NHS Foundation Trust, London, UK; 4 Department of Psychology, University of Bradford, Brandford, UK

**Keywords:** Secondary data usage, Public involvement and governance, Data transparency, Rapid qualitative evaluation

## Abstract

**Background:**

There’s a significant demand to link and analyse administrative and routine local hospital data for health research to improve treatments and understand disease and diagnosis. Involving patients and members of the public in how data are accessed for service improvement is crucial for developing an acceptable, ethical and information governance-compliant whole system data linkage. A key challenge is ensuring sustainable and genuine public engagement that fosters trust in data use. This study evaluates the early implementation of a Data Trust Committee (DTC) at a London hospital, assessing its impact on research efficiency and the experiences of key stakeholders, including patients, staff and researchers.

**Methods:**

A rapid qualitative evaluation was conducted using semi-structured to assess the implementation and perceived impact of the DTC. Purposive sampling targeted DTC members (*n* = 8), attendees (*n* = 3), and researchers (*n* = 2). Thematic analysis, supported by RREAL sheets, identified key themes in stakeholders’ experiences and perceptions.

**Results:**

Findings highlighted five key areas: (1) the programme theory, outlining the DTC’s role in data governance and responsible data access; (2) varying stakeholder perceptions of the DTC’s purpose and decision-making processes; (3) The DTC’s impact on research oversight, data access and approval processes; (4) challenges related to role clarification and communication; (5) the perceived effectiveness of the DTC in enhancing data quality, research oversight and approval speed. While participants recognised the DTC’s potential to enhance data quality and prioritising patient experiences, challenges related to the speed of applications, communication gaps, and technology barriers were identified.

**Conclusion:**

The DTC played a pivotal role in reshaping research regulatory processes, and how this may benefit patients. However, balancing ethical risks with patient benefits remains an ongoing challenge. Addressing role clarity, communication strategies, and stakeholder engagement is essential for optimising future DTC implementation. Future research should expand to evaluate DTC models across diverse healthcare settings to enhance data sharing frameworks.

**Supplementary Information:**

The online version contains supplementary material available at 10.1186/s40900-025-00693-4.

## Abstract (es)

Administrative and routine data are collected by hospitals in their daily operations and used for research and service improvement. A key challenge is making sure patients and the public are involved in deciding how these data are used to ensure it’s done in a safe and ethical way and for patient benefit. This study looked at how a new Data Trust Committee (DTC) was set up at a hospital in London. The DTC was designed to involve patients to make decisions about who can access patient data and how it can be used. We interviewed people involved in the DTC, such as patients, hospital staff, and researchers to understand their experiences. They told us about how the DTC has made accessing data easier, and how it has improved the way data are used for patient benefit and the DTC made sure that patients’ experiences were considered. They also mentioned some challenges, like needing clearer roles for DTC members, improving the speed and communication in the application process and finding the right balance between protecting patient privacy and making sure data are used to benefit patients. In the future, more research should be done to help improve how data are shared and used in healthcare and share this learning. The involvement of patients and the public was important in shaping this study and ensuring that their voices are heard in decisions about how their data are used.

## Public contribution

The aim of this study was to explore the experiences of patients, public and healthcare staff in implementing a Data Trust Committee. Public and patient representatives participated as study respondents and they played an active role in reviewing study materials and shaping our research process. Their contributions helped refine our approach and ensured alignment with the GRIPP2 checklist. Public and patient involvement is central to the DTC’s functioning, our findings revealed that the distinction between patients and public members within the committee was not always clearly defined, leading to ambiguity regarding their respective roles. Patients provided insights based on their lived healthcare experiences, while public members contributed broader societal and ethical perspectives on data access, transparency and the impact of the DTC. Their contributions were central in shaping our understanding of the DTC’s implementation and potential impact on patient care and research ethics. Our findings highlight the importance of refining the DTC’s framework to ensure that both patient and public voices are meaningfully integrated in ways that align with their unique expertise.

## Introduction

Researchers increasingly use large electronic health record (EHR) databases to enhance direct care and treatment of individuals. A significant portion of this work includes secondary data analysis [1], which broadens our understanding of disease causation, associations and prevalence, while addressing both local needs and wider scale issues [2]. From the development of initiatives such as National Programme for Information Technology (NPfIT) [3] to collaborative efforts headed by government bodies and public agencies [4] there has been a significant increase in the number of data use initiatives. Utilising EHRs empowers researchers and institutions to access rich, comprehensive clinical data repositories that include extensive patient records [5]. EHRs help link routinely collected data to ensure greater targeting and cost-effectiveness of interventions, providing a better understanding of diseases and service improvement. This is particularly timely given that public health budgets have decreased by 26% on a real-terms per capita basis since 2015-16 [6]. The growth in EHRs enables the identification on gaps in equity of access, such as health inequalities associated with acute care [7] and wider health service challenges can be identified and actioned [8]. Additionally, linkage and analysis of routine data have the potential to enhance and improve service evaluations, capturing longer-term outcomes.

Despite these benefits, the challenge of data privacy, public trust and being transparent about its operations and limits remains paramount [9]. The concept of Data Trusts has emerged as a potential solution to address these concerns [10], offering a framework for managing sensitive data and allowing it to be shared and used responsibly. Data Trusts are independent participatory governance structures designed to manage data-sharing agreements and ensure data use is aligned with public interest and ethical standards [11]. These trusts allow for the secure and transparent sharing of data, where the public can have confidence that their data is being used ethically for research or public benefit. The UK government’s report ‘Data Trusts: A new tool for data governance’ [12] conceptualised ‘data trusts’ as independent structures for the stewardship of data that enable flexible and inclusive data governance. The underlying model of data trusts seeks to make trustworthy decisions about data in relation to who has access, under what conditions and who it seeks to benefit [13]. However, balancing the diverse goals of stakeholders and ensuring meaningful public engagement in these governance models require further research [9].

When data sharing benefits the public, it can strengthen connected routine data and improve patient care [10]. However, collecting EHR data is costly and time intensive, and researchers face challenges related to the completeness and accuracy of datasets [14]. Additionally, long waiting periods for data access, unclear governance processes, and limited analytical capacity hinder the full potential of routine data use [15]. Addressing these issues requires robust information governance frameworks and greater transparency in data-sharing protocols to prevent project failures and inefficiencies.

Recent initiatives highlight the role of Data Trusts in addressing challenges around transparency and ethnical use of data. For example, the PIONEER Hub led a series of public and patient events, serving as a framework for encouraging the use of health data [10]. Data Trusts are central to this process by ensuring that data sharing is managed responsibly, directly addressing public concerns about data privacy and ethical use. These frameworks rely on Data Trusts to ensure that data access aligns with public expectations of privacy, ethical use, and transparency. The *Unlocking Data* protocol [16] by East Sussex County Council and Kent County Council also outlined the need for structured public involvement in dataset governance. Similarly, research by HDRUK into public attitudes towards the use of routine data found that members of the public wanted to be involved in making decisions about whether the public good was being served [15]. However, the biggest challenge facing the use of public data is trust from members of the public and patients, in their data being anonymised, accessed and used responsibly, and non-exploitation [17].

A Data Trust Committee (DTC) differs from a Data Trust in that it is a more focused governance body operating within an institution or organisation, often responsible for overseeing data access requests and ensuring ethical decision-making at the point of use [9]. The DTC for this study was at a London hospital [18] set up to include patients in the governance and approval process for applications within a large teaching hospital [19]. The DTC initiative aimed to develop a deeper understanding of public perceptions of data use and ensure that patient data is handled responsibly. The DTC plays an integral role within the Data Access Process (DAP-R), providing oversight, ensuring transparency, and conducting ethical reviews of research projects. Comprising both patient and public representatives and hospital staff, the DTC fosters collaboration between patients, the public, the NHS, universities, data scientists, and industry partners [18]. However, it is essential to distinguish between the roles of patients and lay public members. While patients provide direct experience, lay members offer a broader societal perspective, ensuring balanced decision-making that represents both individual and public interests [20]. Furthermore, the distinct feature of the DTC model is the inclusion of staff members who act as patient advocates.

**Fig. 1:**
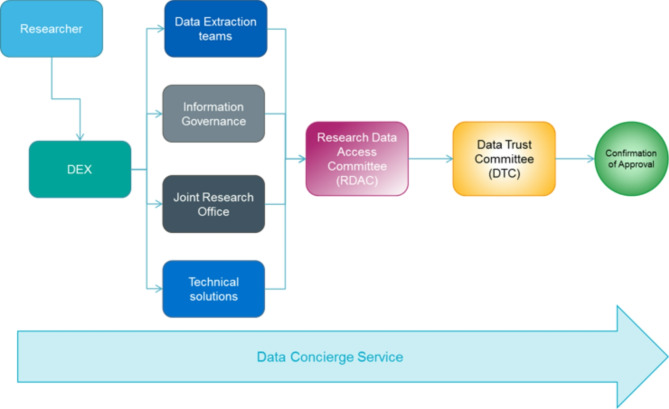
DAP-R: A stage process

Once the request for data request is finalised, it proceeds to the DTC as the final step in decision-making, where they either approve or decline the request. The DAP-R system (see Fig. 1) enables hospitals to grant researchers access to data without requiring a separate HRA/REC submission. The DTC model aligns with the Five Safes framework [21], developed by Office of National Statistic (ONS), which provides best practice for balancing data protection with open science principles. This process is used specifically for studies involving anonymous data and do not require any personally identifiable information. Researchers submit data access requests through the Data Exchange (DEX) system, with the Data Concierge service providing guidance throughout the process. The DTC reviews each request and reaches a consensus on whether to approve the data request [10].

This governance model aligns with a previous study [17] involving patients, carers, healthcare staff, and the public, which found that sharing data without patient benefit was a major concern among participants. The study’s recommendations emphasised supporting data use when there are clear benefits to NHS patients or the wider population, and establishing NHS oversight as the most trusted organisation. Additionally, the NIHR-funded Whole System Data Linkage Accelerators to unlock public health data [22] project found that systems ensuring ongoing clarity, transparency, and accountability to the public and researchers could help improve awareness and understanding. Data Trusts and DTCs provide complementary approaches to ethical data governance, reinforcing the importance of structured, participatory oversight in health data use.

### Aims

The aim of this study was to explore how the programme theory underpinning the implementation of a DTC was applied during its early stages, focussing on stakeholders’ perceptions and experiences of the DTC’s role in facilitating responsible data access. Additionally, it aimed to evaluate the challenges and opportunities associated with public involvement and engagement in data-intensive health research.

The rapid evaluation was guided by the following questions:

1. What is the purpose of the DTC as perceived by stakeholders?
2. What is the perceived impact of the DTC on:
  - The speed of the research processes (i.e., study setup, data access and approval speed).
  - The utilisation of data.
  - On DTC members.
  - On researchers.
3. What aspects of the DTC are working well?
4. What lessons can be learned to inform future planning and implementation?
5. What is the underlying programme theory supporting the DTC?

## Methods

### Design

We conducted a rapid evaluation, with interviews as the source of data to assess the implementation and perceived impact of the DTC [23]. This study design focused on interviews to enable data to be collected and analysed in a targeted manner, under limited timeframes [24]. We conducted data collection and analysis in iterative cycles, proceeding concurrently to inform subsequent data collection stages [25].

### Sample and recruitment

Purposive sampling was used to recruit members and attendees involved with the DTC. Due to its recent establishment, the DTC had a restricted number of participants. Participants were contacted by one of the researchers (SM) or were introduced to the evaluation by one of the DTC attendees. Participants emailed one of the researchers directly, expressing their interest in taking part. Interviewees were then sent the participant information sheet and consent form. Participants provided informed consent before the interviews to ensure that they were happy with the inclusion of their data.

### Data collection

Data collection was conducted between April and June 2023 and carried out using semi-structured interviews over Zoom and Microsoft Teams. Interviews were conducted by four researchers working in parallel, using an interview topic guide based on the evaluation questions guiding the study. The average length of interviews was 31 min, with a range from 17 to 48 min. Interviews were audio recorded and subsequently interview notes were entered into RREAL sheets to capture the key summaries of the discussions, highlighting relevant points and reflections, while verbatim quotes were used for illustrative purposes in presenting specific participant views. The semi-structured interviews aimed to capture participants’ perceptions of the committee’s purpose, its impact on research and individuals associated with the DTC, successful aspects, and areas for improvement. (See appendix 2 for interview the topic guide). Those affiliated with the DTC discussed their committee-related experiences, while researchers shared their experiences with the DTC application.

### Data analysis

We employed rapid qualitative data analysis methods to analyse interview notes and recordings. This process involved a systematic, team-based approach to develop and refine themes. To ensure consistency, we began by developing an initial coding framework, informed by the study’s evaluation question, prior literature on data trust governance and early familiarisation with the data. We identified initial codes from interview notes and recordings using RREAL sheets, which were organised in rows and columns (see Appendix 3 for RREAL sheet with notes) [26] to capture key responses and patterns across participants. Codes were generated deductively based on the evaluation questions, and inductively from emergent participant narratives to shape the coding structure. To ensure rigour, the evaluation team met weekly to review and refine the coding framework, discussing overlapping or ambiguous codes, the indexing of codes and merging or splitting themes as necessary. This iterative process allowed us to refine themes as new data was collected. Themes were then consolidated by grouping related codes into broader categories that captured the key insights related to the experiences of the DTC. RREAL sheets served as a dynamic tool throughout data collection and analysis, facilitating the synthesis of information across the entire data set [26]. This enabled both the identification of gaps in data and data saturation when no new information emerged [27]. The RREAL sheet guided our in-depth analysis and collaborative interpretation of the data to address our overarching research questions, validating themes and minimising bias.

For this evaluation, we employed separate RREAL sheets for each respondent group (see Appendix 1 for an example of the RREAL sheet) [26]. Following the Rapid and Rigorous Qualitative Data Analysis (RaDAR) technique [28], we systematically reduced the volume of the qualitative data into concise summaries. The iterative refinement process known as ‘data reduction’ transformed the depth and richness of the thematic data into a more manageable and user-friendly format [28]. This was facilitated using RREAL sheets, which helped to organise the summaries and further streamline the data for analysis. The RREAL sheets acted as a tool within the RaDAR technique to capture key themes and ensure that the information remained accessible and actionable. Additionally, we conducted a documentary analysis of informal documents and website information related to the DTC, including publicly available resources such as policy documents, guidelines, and relevant website content. This approach enabled us to incorporate information not covered in interviews, thereby deepening our understanding of the broader DTC application process and addressing knowledge gaps.

### Ethical review and governance

The study was classified as a service evaluation by the HRA decision tool. The research team followed ethical principles for the conduct of the research throughout the study, including voluntary participation, informed consent, de-identification of research participants, confidentiality of data and the opportunity to withdraw from the study. All researchers had been trained in research ethics and information governance.

## Results

We identified five key findings related to the evaluation questions guiding this study: (1) programme theory outlining expected outcomes, and necessary steps for successful implementation; (2) stakeholder perceptions of the DTC’s purpose and decision-making processes; (3) perceived impact of the DTC; (4) areas of effectiveness and (5) challenges and improvements. The findings are presented below thematically and as per RaDAR techniques [28].

The sample (Table 1) included DTC members (patients and staff who were clinicians *n* = 8), DTC attendees (who were part of the management of the committee, but not part of the decision-making for the applications *n* = 3), and researchers (who had applied to applications using the DTC *n* = 2).

**Table 1:** Participant types

Participants	*N* =
DTC members	8
DTC attendees	3
Researchers	2
Total	13

### Programme theory

**Fig. 2:**
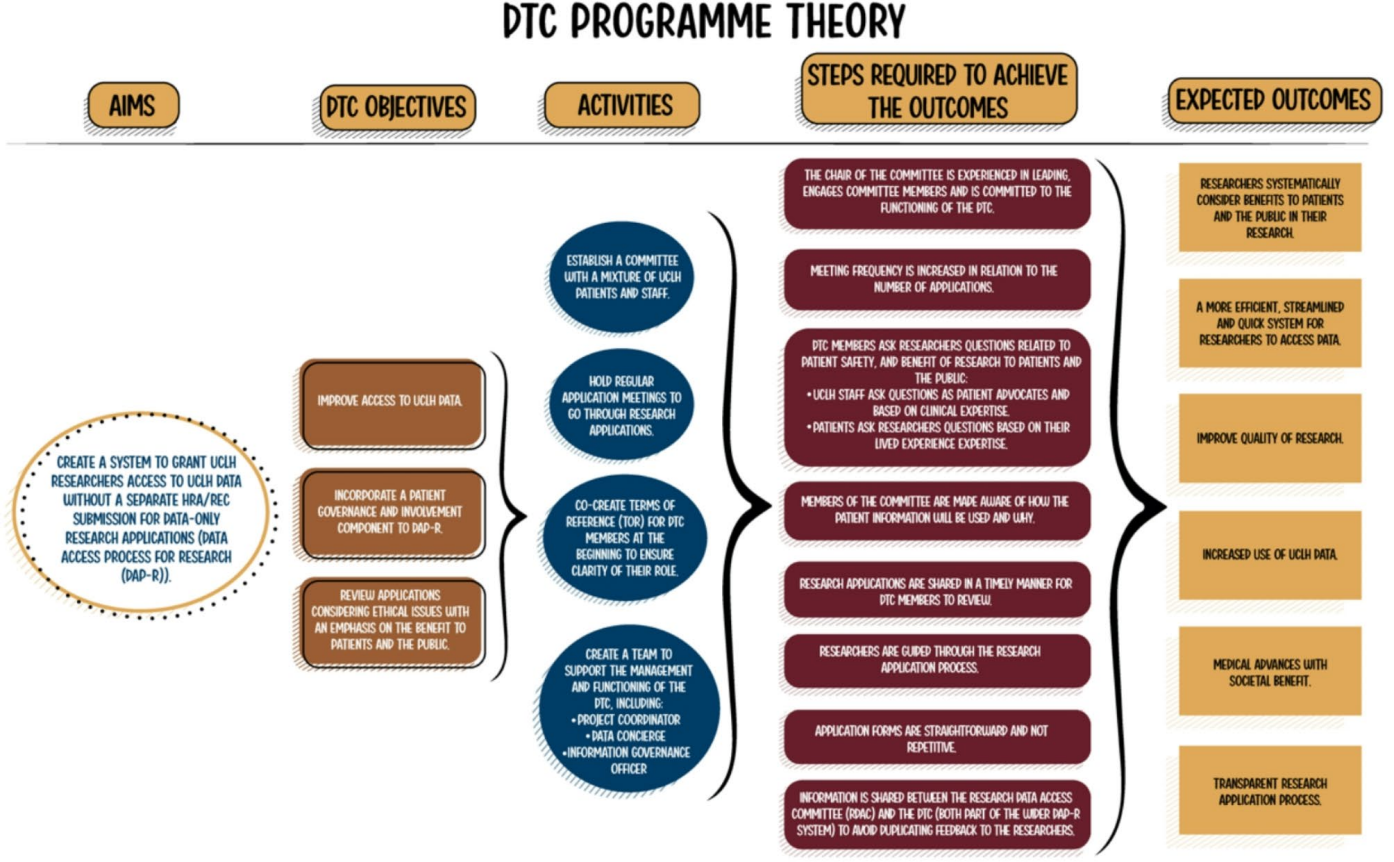
DTC programme theory

A programme theory was developed to outline the aims, expected outcomes and necessary steps towards achieving these outcomes (see Fig. 2). These steps encompass both ongoing activities and factors contributing to achieving the outcomes, incorporating suggestions for improvements provided by participants. This programme theory serves as a model framework, offering guidance for the establishment of other DTCs.

The DTC Programme Theory establishes a structured system to grant researchers streamlined access to data while embedding ethical governance and public involvement. Its objectives include improving data accessibility, incorporating patient and public perspectives in decision-making, and ensuring ethical scrutiny of applications. The DTC achieves this through a committee composed of staff and patients, regular meetings, and clearly defined roles. Members review applications, address ethical concerns, and ensure transparency in data governance. This process results in a more efficient research application system, higher-quality research, increased data utilisation, and strengthened public trust, ultimately leading to medical advances with societal benefits.

### Stakeholder perceptions of the DTC’s purpose and decision-making

The perception among DTC members and attendees of the DTC’s purpose encompassed distinct views on its intended role and impact on the research process.

### Ensuring data access through inclusive and transparent processes

Participants perceived that the main objective of the DTC was to have a broad range of stakeholders to approve access to data for data-only studies. Patient members were seen as being a crucial role in data governance, ensuring that data governance remained person-centred. According to patient participants, the inclusion of staff members, was seen as providing valuable advocacy on their behalf and offered clinical perspective to complement their viewpoint.

“*Clinicians bring a more clinical perspective, whereas patient members have a different perspective which bring helpful insights*” (DT_12, DTC members).

The DTC was designed to ensure that researchers, guided by patient benefits, maintained transparency and conducted high-quality research. The assumption was that this approach would result in research outcomes that directly or indirectly benefitted patients and the public, fostering a culture of accountability.

*“All projects will have patient involvement, makes researchers think about patient needs in design, this will have broader purpose of show”* (DTC14, DTC attendee).

### Ethics-focused review of applications

Many DTC members and attendees indicated that the key function of the DTC was to determine whether research applications were ethical, with a particular emphasis on the benefit of patients and the public. Factors mentioned in this context included patient safety, confidentiality, usefulness, quality, and feasibility within the given setting.

*“Our role involves determining if research is fit for purpose […] will it have benefits for the patients overall in respect of our population?*” (DT_08, DTC member).

DTC members considered their role as essential in deciding whether projects were ethically sound and evaluating the appropriateness of projects, similar to NHS research ethics committees. One participant explained *“essentially doing what an NHS REC or RA committee would do*,* like an ethical review to look at the appropriateness of a project”* (DT_12, DTC member).

Members prioritised ethical data handling, particularly in collaboration with private companies, ensuring that proper governance agreements were in place in addressing concerns about these agreements:

*“What’s going to happen with the data? Who owns the data? Who owns the IP of the project?” (DT_05, DTC member).*

### Improve access to data

Another theme frequently discussed was the DTC’s role in improving data accessibility and efficiency in the research application process. DTC members expressed confidence that the DTC design was intended to reduce bureaucratic hurdles, clarify expectations for applicants, and streamline the approval process.

*“The applications will improve*,* they’ll be clearer*,* and the critical elements will be more obvious… applicants will know what they are expected to provide”* (DT_09, DTC member).

The shift from a model requiring navigation through multiple approval bodies to a more efficient process was emphasised by participants, who described it as a *“streamlined approach”* (DT_08, DTC member, ST) and *“more efficient”* (DT_15, DTC attendee). The DTC’s focus on researcher satisfaction and support for early career researchers was also highlighted, with one member noting “*we want researchers to be happy and to be clear about how they carry out their research”* (DT_06, DTC member) and an example of a coaching session for a PhD student.

The researchers viewed the DTC as an agile application process, a *“one-stop shop”* with diverse stakeholders capable of delivering timely approvals. However, documentary evidence suggests that streamlining applications was not part of the original purpose of the DTC. The findings suggest variance in perceptions regarding the DTC’s purpose, the extent to which these priorities align in practice remains an open question, highlighting an enhanced approval speed could eventually evolve as a natural outcome of the DTC’s operational processes.

*“Kind of an ideal scenario*,* one stop shop. To get your process of your project approved”* (DT_17, researcher).

### Role of DTC members

Many members of the DTC expressed a sense of ambiguity regarding their roles at the outset, and that these roles gradually took shape as the process unfolded. However, as the committee took form, the roles of the DTC members were collectively defined, alongside the establishment of the Terms of Reference. This iterative process suggests that governance structures within the DTC were co-produced rather than imposed, allowing members to define their contributions. Despite this, members clarified that they are not the ethical approval body, addressing a common researcher misconception equating DTC approval with ethical clearance.

*“I didn’t feel ready in the sense I didn’t know what to expect, but I didn’t hear anyone did and we kind of went along with it and we agreed on the terms of reference together in our first couple of meetings. And so, we created the role along with obviously the people that created the concept of the DTC*” (DT_03, DTC member).

The collaborative nature of the of the DTC was widely recognised by members. They believed that their input on applications effectively incorporated diverse ideas and experiences, thereby enhancing the robustness of the decision-making process.

*“The staff and patient representatives are all, like, very different, very diverse and all bring different ideas to the table*” (DT_14, DTC attendee).

However, while diversity in membership was seen as a strength, differences in role expectations persisted, particularly regarding staff members’ contributions. Clinical expertise was acknowledged as helpful in clarifying medical and clinical terminology used in applications. However, there was an implicit distinction between the types of questions raised by professionals and those posed by patient or public representatives.

*“A diverse group […] some with medical knowledge. They asked questions, medically related, but the patient people like myself, […] we might think of other questions that maybe as a patient, the others wouldn’t ask*” (DT_06, DTC patient member).

### Perceived impact of the DTC

As a relatively new entity, the DTC’s impact was perceived as emerging and in the process of generating impact. DTC members believed the robust application process prompted researchers to present proposals with greater scrutiny of data use and anonymity, signalling a shift towards more responsible data stewardship, oversight procedures, review and approval speed. The primary role of members was to review applications with a focus on patient confidentiality and ultimately decide if an application *“will benefit patients… and ultimately to decide whether the application goes ahead*,* needs amendments or to be resubmitted”* (DT_04, DTC attendee).

*“I think that’s quite valuable when they present their research*,* you can see that they’re thinking about […] our choice of words and respect of the data that they’re trying to capture and that they’re changing the way in which they then present their proposal in respect of the data”* (DT_08, DTC member).

While some researchers viewed the DTC a route to access data and a procedural step. DTC members saw it as a mechanism for shaping data governance. This distinction underscores differences in perceptions between administrative and ethical oversight.

“The use of the data is defined by the scope of the project and so if you’re applying with a sort of predefined data management analysis plan, it shouldn’t really change anything. It’s more of a route to access of the data” (DT_18, researcher).

DTC members, particularly patient members highlighted that their involvement helped them feel more connected at an organisational level, contributing to their sense of belonging within the wider research ecosystem. This awareness is particularly beneficial for patient members, who gain an enhanced understanding of research processes, shifting their perception of data governance from sceptics to confidence, reinforcing trust in data sharing practices for patient benefit.

*“Being part of the DTC has definitely made me feel more confident about how data is used. Before, I had concerns about what happened with patient data, but now, seeing the process and being involved, I feel reassured that it’s being handled properly and for the right reasons*” (DT_12, DTC member).

### Perceived impact on oversight procedures and guardianship of data

DTC members and attendees believed the DTC had strengthened oversight procedures and data guardianship process has *“added a layer of reasonability to the application and increased the quality*” of how data governance is managed (DT_03, DTC member). They saw its ethical input improved the quality of applications and ensured a more consistent and transparent approach, particularly valuable in protecting intellectual property, guardianship of anonymous data, more consistent utilisation of datasets, and visibility.

*“I think they probably have a stronger impact when it comes to guardianship because you know*,* we have information governance and we have legal responsibilities. […] I would say they’re probably enhanced that feeling of guardianship over anonymous data compared to what it was or what it would be without the DTC”* (DTC_15, DTC attendee).

Some participants felt the DTC helped change the perception of risk in the hospital and the research process by embedding patients and public within governance structures, the institution was able to demonstrate a transparent and patient-governed process. DTC members mentioned that the trust needed to show diverse participation in their data governance structures.

### Impact on speed of review and approval

While some members thought the DTC had improved the speed of review and approval processes, others believed it would inevitably increase the amount of time it took to process projects. However, a key factor influencing this was a requirement for more robust applications, which meant some high-quality applications would be streamlined and would move quickly through the process, while others would need to be resubmitted for quality and detail before they could be approved which meant they faced delays.

*“We thought that was all a speeding up process of approvals*,* but it turned out that you had to still go through the JRO after you got DTC approval […] Now we were happy to go through all that. […] if it meant that what we did would then streamline every study following us afterwards”* (DT_17, researcher).

Despite concerns about potential delays, some participants felt the DTC reduced red tape for the researchers. Participants acknowledged the committee was still in its early stage and with time would become more streamlined. Researchers were split about the speed of review. While one found it slow, another saw it as the most straightforward and transparent application processes.

*“This was overwhelmingly the most straightforward*,* most transparent*,* and best of them […] from a speed perspective*,* it surely has to be some of the best”* (DT_18, researcher).

### Impact on those involved with the DTC

The involvement with the DTC had diverse implications for DTC members and researchers in relation to their roles and experiences within the committee’s framework.

### On DTC members

Many DTC members found their participation to be highly positive and rewarding. They described the experience as refreshing, enjoyable and creating a sense of fulfilment in terms of making a real difference to research governance. Members felt they had a better understanding of research processes, including the role of the NHS, ethical considerations, and the challenges researchers face. Members valued the opportunity to engage with real-world research projects, which deepened their appreciation for the complexities of data governance, patient confidentiality and ethical considerations and the challenges researchers face. *highlighted* that because everyone had different expertise and perspectives, they were able to learn from each other.

*“I definitely have found it very interesting*,* informative. I love hearing all the […] novel things that are being trialled. And I think it’s very interesting for me as a clinician to have patient members on the panel and hear their take on things […] from a personal point of view*,* I’ve learned a lot”* (DT_03, DTC staff member).

A key benefit of involvement was the opportunity for collaboration and shared learning. Members highlighted how working alongside individuals from different backgrounds and expertise *“Members learn from one another’s perspectives*,* fostering a deeper understanding of research processes*” (DTC_16, DTC member).

### On researchers

DTC members believed that the comprehensive feedback provided had a positive impact on researchers. This improved their requests, ensuring better ethical considerations, rigour and quality of research proposals. Although some researchers initially found the process challenging, particularly if they did not immediately have their research approved, they recognised the value of the feedback in strengthening their applications. Researchers who successfully modified and resubmitted their applications acknowledged that the process improved their research quality and felt positive about doing a similar project again and that they would engage with the DTC again.

*“This feedback allows researchers to make necessary amendments, leading to stronger proposals”* (DT_03, DTC member).

### Areas of effectiveness

The evaluation uncovered key areas where the DTC demonstrated effectiveness and efficiency (Table 2), shedding light on its successful aspects within the research application and approval processes.

**Table 2:** Areas of effectiveness

Area of effectiveness	Key Points	Quotes
DTC Meetings	-DTC members and attendees praised the committee for dedication, attentiveness, and collaboration. - Meetings were well-organised with regular attendance and focus on progress. - Multidisciplinary nature contributed to balanced decision-making. - Researchers appreciated the opportunity to present projects and the enthusiasm from DTC members. - Inclusion of patient perspectives on data governance were seen as valuable and meaningful.	“It was quite enjoyable, I was really able to tell them what we were doing, why we were doing it. It was nice to hear patients being very enthusiastic about it” (DT_17, researcher).
The Role of the DTC Chair	- Widely regarded as essential for committee’s effectiveness. - Chair was approachable, supportive, and well-suited for the role. - Encouraged an environment where everyone could express their views and reaching collective decisions. - Promoted mutual respect and collaboration within the committee.	“The chairman is very, very approachable, very easy to talk to” (DT_06, DTC member). “The relationship and the interaction between the members of the DTC and the Chair is just quite good *[…]* people are able to give their views. Everyone is” (DT_14, DTC attendee).
Preparation for Meetings	- Acknowledged for their helpfulness in supporting the meetings. - Ensured smooth functioning by managing administrative tasks. - Members appreciated having ample time to review papers before meetings, improving discussion quality. - Project coordinator ensured timely distribution of agendas, managed logistical arrangements and supporting documents. - Highly valued for responsiveness, knowledge, and problem-solving during the application process.	“It’s already very, very well-planned process.” “We’re sent an e-mail by [name], the [person] that sort of organises everything, a good few days before the actual meeting” (DT_06, DTC member).

### Challenges and improvements

The main themes regarding challenges and suggested improvements to the DTC process included: time taken to review applications for approval, the application process, the communication between the RDAC and the DTC, and technological challenges (Table 3). These themes highlight a number of lessons learnt that can inform the future functioning of the DTC. As the evaluation was carried out alongside the rollout of the committee, some of these challenges and improvements have already been addressed.

**Table 3:** Challenges and improvements for the future functioning of the DTC

Theme	Challenge	Suggested improvements
**Time taken for review of applications**	Overall approval processes were time-consuming, causing **researchers** to caution that project approvals might coincide with contract expiration or money for projects may have run out. Monthly meetings raised concerns regarding the DTC’s scalability in managing applications for large research institutions.	**Researchers** proposed the idea of having more frequent application review meetings. **DTC members** suggested a larger committee could meet on a rota and accommodate increasing demand. **Researchers** suggested streamlining the process by grading applications according to complexity. **Researchers** recommended pre-discussion of applications outside the meeting and proposed limiting the number of members present (three or four) during meetings to speed up the review process for each application.
**The application process**	The DTC occasionally lacked the expertise to approve certain applications.	**DTC members** suggested providing applicants with comprehensive guidance on presentations and the application forms. **DTC members** and attendees requested the creation of a detailed protocol outlining the necessary elements for improvement within an application. **DTC members** requested exploration into methods for accessing the appropriate expertise where the necessary expertise was not present within the committee.
**Communication between the RDAC and the DTC**	**DTC members and attendees** highlighted concerns about the overlaps between RDAC and DTC, citing challenges in acquiring accurate information for application assessments. These issues were linked to communication difficulties between DTC members and the applicants.	**DTC members and attendees** expressed an interest in better understanding of each stage of the research application process. They proposed attending some RDAC sessions or gaining access to their handover or discussion sheets for better insights. **DTC members** requested further transparency and visibility between the RDAC and DTC, advocating for shared feedback visibility to prevent duplication between committees.

## Discussion

Our findings reveal that the DTC serves multiple functions, including ethically reviewing applications and ensuring patient and public benefits, with varying perceptions of its broader roles. The DTC’s conceptual foundation drew inspiration from similar models like the PIONEER Health Data Hub. Members highlighted the importance of reshaping research processes and the impact on individual lives. However, previous research highlights patient concerns about data sharing without direct benefit to individuals, and the ethical use of data is more likely to gain support when it directly benefits NHS patients, with greater trust in NHS data use [17, 29]. This reinforces the importance of focusing on both patient and societal benefits, which emerged as key factors in determining the ethical and acceptable use of data [17]. The DTC fostered transparency in data use through an open dialogue with the public and patients, as recommended by the PIONEER hub [10].

The distinction between patient and public perspectives in evaluating data use is crucial so that DTC members understand their specific roles. Patients often focus on the direct benefits to individuals with lived experience of healthcare, whilst public perspectives can provide insights into societal benefits. It remains unclear if DTC members were explicitly guided in distinguishing their perspectives. Future iterations of the DTC, or similar committees, should offer clearer definitions to ensure members have relevant knowledge and make their contributions more meaningful [30]. This distinction also raises broader questions about power dynamics and authority within the DTC. While medical expertise ensures technical clarity, patient perspectives bring vital lived-experience insights that challenge assumptions and broaden ethical considerations. The balance of these contributions in decision-making warrants further investigation. Our findings suggest that the DTC operates as a site of negotiated governance, where roles and responsibilities evolve through ongoing dialogue. Although this issue was not explicitly addressed by the DTC to date, future evaluations should ensure that the roles and expectations of patient and public members are clearly defined and communicated [31].

The inclusion of medical and clinical staff members was beneficial due to their expertise, which patients felt they lacked. This highlights the challenges that DTC members experience in balancing the ethical risks against potential patient benefits [32]. Despite the positive impact of including medical and clinical staff, it is not the role of lay members to judge the technical quality or relevance of the research. Instead, lay members need a clear understanding of their expected contributions and sufficient background information to perform their role effectively. Future training should focus on judging the quality of involvement processes and conducting ethical reviews, rather than assessing technical aspects of the research. The role of the chair was pivotal in supporting public and patient members by highlighting research risks during decision-making, fostering a supportive environment where staff can guide patients and the public in assessing risks while emphasising the need for public benefit [30, 32, 33].

There were differing perspectives regarding data access and the way data were used. Despite the inclusion of the public and patients, there may still be limited trust in the process as being genuine regarding its objective to increase public good [34]. Recent evidence has found that despite patients’ willingness to share data, concerns around mistrust regarding how data is protected and used persist. The involvement of public and patient members in the design and conceptualisation of ethical and institutional processes to approve data access is imperative to address these concerns. Furthermore, the inclusion of members from ethnically diverse and socioeconomic backgrounds may help facilitate increased data sharing among these groups, considering these groups are known to experience lower levels of sharing data [35].

A significant challenge identified in our study was the differing perspectives on data access. Researchers focused primarily on the need for timely access to data, highlighting challenges around accessing data in a timely way and the need for additional feedback. A common concern in using EHR data pertains to the complex and lengthy application procedures, significantly impacting on the timely acquisition of data [36]. The DTC should collaborate closely with researchers to establish more efficient methods for obtaining and accessing EHR data. Although the streamlining of application processes is outside of the DTC’s remit/objectives, the findings of this study aid future iterations by addressing these challenges. This suggests there needs to be an increased understanding of data usage among the public and healthcare providers to maximise the potential benefits of research which is used to benefit the public [37]. It is crucial to educate researchers about the use of health data for research and planning purposes [17].

Finally, our findings highlighted that patient members felt more comfortable and trusting of the data-sharing process when they had an active role in reviewing applications and engaging in discussions. This involvement gave them a sense of transparency and control. However, trust in data usage still emerged as a critical concern, despite public and patient involvement, there remain issues around mistrust, particularly in terms of how data is protected and used. This aligns with recent findings indicating that patients are often willing to share data but remain cautious about privacy and security. Addressing this mistrust, particularly among ethnically diverse and socioeconomically disadvantaged groups, is essential to increasing data-sharing rates and ensuring that data-driven research benefits the public.

### Limitations and future research

The findings of this study should be considered in relation to its limitations. The DTC had been in operation for one year at the time of the study resulting in a limited number of researchers having undergone the application process and consequently restricting the number of researchers to recruit from. Initially, the plan involved the inclusion of the local research office staff and designers involved within the DTC. Unfortunately, due to the limited number of local research office staff, alongside the rapid nature of the study, recruiting staff became unfeasible. Additionally, the involvement of four different interviewers may have introduced slight variations in data collection, which could have affected the consistency of responses across interviews.

This study was conducted as a rapid qualitative evaluation and provides a snapshot of the early stages of the DTC during the study period. However, to capture changes over time and the DTC’s long-term impact, future evaluations are necessary. A broader scale evaluation is recommended to capture the views of a more extensive pool of researchers seeking access to hospital data and capture change in attitudes towards data use. Additionally, it is recommended that the evaluation consider the larger DAP-R system that the DTC is a part of, to better understand the DTC’s influence in comparison to other parts of the system (such as the RDAC) on data access. Furthermore, a mixed methods study is recommended to understand the quantitative impact of the DTC on research processes, including the access and use of hospital data.

## Conclusion

This study explored the establishment of a DTC at a London hospital to facilitate the ethical approval process for applications to access patient health data for research. The DTC framework was established to prioritise the inclusion of public and patient voices in the decision-making process around the tangible benefits of data access and using data for patients and society benefit overall. Incorporating staff with medical and clinical expertise enhanced the ethics-focused review within the DTC. However, there is a crucial need for members to be adequately prepared and receive training on their expected contributions and have sufficient background information to perform their role. Despite multiple challenges supporting access to data, this rapid qualitative evaluation reveals a positive impact on members, ultimately improving data utilisation for the public good.

## Electronic supplementary material

Below is the link to the electronic supplementary material.

Supplementary Material 1

Supplementary Material 2

Supplementary Material 3

## Data Availability

No datasets were generated or analysed during the current study.
