# Supplementary material for: Exploring the implementation of a data trust committee: a qualitative evaluation of processes and practices
Source: Res Involv Engagem. 2025 Mar 6;11:19. doi: 10.1186/s40900-025-00693-4 (PMC11887347; doi:10.1186/s40900-025-00693-4)
Supplement: Supplementary file 1 — Supplementary Material 1 [file 40900_2025_693_MOESM1_ESM.docx]

| **Category** | **Findings** |
| --- | --- |
| Background – reasons as to why the DTC was created |  |
| Thoughts on whether it is functioning as intended |  |
| Hope for outcome of DTC |  |
| Description of experience with the DTC |  |
| Benefits in the change that DTC will bring |  |
| Potential risks of DTC |  |
| Perception of DTC impact on research |  |
| Perception of influence of DTC on how data are used |  |
| Perception of whether DTC has changed oversight/ guardianship of patient data |  |
| Perception of impact on speed of review and approval of application |  |
| Perceived impact on DTC members |  |
| Perceived impact on researchers |  |
| Description of any difficulties encountered with functioning of DTC |  |
| Suggestions of how to overcome difficulties |  |
| What works well in DTC |  |
| Contributing factors which help the DTC to work in this way |  |
| Additional comments |  |

Designers – DTC RREAL sheet
