# Supplementary material for: Exploring the implementation of a data trust committee: a qualitative evaluation of processes and practices
Source: Res Involv Engagem. 2025 Mar 6;11:19. doi: 10.1186/s40900-025-00693-4 (PMC11887347; doi:10.1186/s40900-025-00693-4)
Supplement: Supplementary file 2 — Supplementary Material 2 [file 40900_2025_693_MOESM2_ESM.docx]

Topic Guide for DTC members and attendees

Thank you for agreeing to take part in this interview. I am from the Rapid Research Evaluation and Appraisal Lab at UCL, and we have been invited to evaluate the Data Trust Committee.

We are conducting interviews with those who have been involved with the DTC to hear about your experience, and your views on what works and what could be improved. This will help future functioning of the committee.

Your participation in this interview is voluntary. If there is any question you don’t want to answer, that is fine, we will just move on. If you need to stop or have a break for any reason, please let me know, that is completely fine.

The interview will be recorded. What you say in this interview will remain confidential, you won’t be identifiable in our findings or reports. Do you mind if we record the interview on Teams? The video won’t be used for anything, but if you would prefer we can turn our cameras off?

The interview should last around 30 minutes.

Do you have any questions before we begin?

**Background of DTC**

1. What can you tell me about the purpose of the DTC?
   - Do you feel that it is functioning in this way?
   - What do you expect to be the outcome of the DTC?
2. Do you mind telling me a bit about your involvement with the DTC?
   - How would you describe your role?
   - Has it been what you expected?
   - What is the role of the DTC members? (***DTC attendees only***)
   - Did you feel prepared for the role? (***DTC members only***)
3. Overall, how would you describe your experience with the DTC?
   - How do you find the experience of having a committee that includes both staff and patient members?

**Perceived impact of DTC**

1. What would you say are the benefits in the change that the DTC will bring?
   - Can you tell me more about these?
   - Do you think that there are any risks?
2. Do you feel the DTC has had any impact on research?
   - Do you feel it has had an influence on how data are used?
   - Do you feel it has changed the UCLH’s oversight procedures/guardianship of patient data?
   - Do you feel it has had an impact on the speed of review and approval of applications?
3. Do you feel the DTC has had an impact on those involved, whether negative or positive? (*If clarification is needed*- impact relating to being part of the committee, or to having to go through the committee for applications)
   - The DTC members?
   - The researchers?

**Improvements and what is working well**

1. Have you encountered any difficulties in your time being on the committee?
   - Can you tell me a bit about these difficulties?
   - What do you think would help overcome these difficulties?
   - (*If no difficulties are mentioned)-* Do you have any suggestions for improvements?
2. What do you think works well with the DTC?
   - What do you think helps it work in this way?

**Closing**

1. Is there anything that you would like to share that I haven’t asked you today?

Topic Guide for Researchers

**Background of DTC**

1. What can you tell me about the purpose of the DTC?
   - Do you feel that it is functioning in this way?
   - What do you hope to be the outcome of the DTC?
2. Overall, how would you describe your experience with the DTC?
   - How was the application process?
   - Did you find it helpful? What did you find particularly helpful/unhelpful?
   - How does it compare to other experiences you have had applying to use data from other committees?
   - Knowing that your application would be reviewed by the DTC, did you adapt your application in any way?

**Improvements and what is working well**

1. Have you encountered any difficulties with the functioning of the DTC?
   - Can you tell me a bit about these difficulties?
   - What do you think would help overcome these difficulties?
2. What do you think works well with the DTC?
   - What do you think helps it work in this way?

**Perceived impact of DTC**

1. What would you say are the benefits in the change that the DTC will bring?
   - Can you tell me more about these?
   - Do you think that there are any risks?
2. Do you feel the DTC has had any impact on research?
   - Do you feel it has had an influence on how data are used?
   - Do you feel it has changed UCLH’s oversight procedures/guardianship of patient data?
   - Do you feel it has had an impact on the speed of review and approval of applications?
3. Do you feel the DTC has had an impact on those involved?
   - The researchers?

**Closing**

1. Is there anything that you would like to share that I haven’t asked you today?
