# Supplementary material for: Exploring the implementation of a data trust committee: a qualitative evaluation of processes and practices
Source: Res Involv Engagem. 2025 Mar 6;11:19. doi: 10.1186/s40900-025-00693-4 (PMC11887347; doi:10.1186/s40900-025-00693-4)
Supplement: Supplementary file 3 — Supplementary Material 3 [file 40900_2025_693_MOESM3_ESM.docx]

RREAL Sheet for DTC members and attendees

- ST = staff member
- PT = patient member

| **Category** | **Findings** |
| --- | --- |
| Perception of purpose of the DTC (why it was rolled out, expectation for outcome) | - Act as the delegated function of the HRA (DTC_15, DTC attendee), an HRA devolved process (DT_12, DTC member, ST) - In future if it is working in a in a way that was intended, the DTC might also be able to be consulted on some more broad issues around how they use data at a senior management level (DT_14, DTC attendee). - With the proliferation of massive data, it has more important to have more control over how data access is requested, how is secured and accessed. Then, the purpose of the DTC is to make sure that the data that's being used is being used ethically, but also that the research projects are indeed providing some patient and public benefit (DT_05, DTC member, ST). - It's been set up as part of the purpose to establish UCL as a leading World Class Research Hospital and it that's a sort of global purpose. It's all the purposes also for the people involved in it to improve patient safety, the quality of the service, and to be able to do this much more rapidly because we're using, existing data and very recent data, and there isn't the usual long time scale (DT_02, DTC member, PT).   Diverse committee incorporating patients and public   - With fully digital health record, wealth of data people are interested in using. DTC created as a final common pathway for approval to access of data for data-only studies, with a generalist approach, which is why it is created with clinicians and patient representatives (DT_3, DTC member, ST) - Allows good general understanding of research applications as comprises of different perspectives (combo of staff and public), from different stakeholders about the implications of that particular research (DT_08, DTC member, ST). - I think that part of the outcome in that sense will be the contribution to the overall governance of the trust because they must be able to demonstrate high levels of patient participation and involvement and the DTC can contribute in that way (DT_02, DTC member, PT).   Ethics- focused   - Creating a scrutiny panel in respect of the research applications to determine if application is ethical (DT_08, DTC member, ST), ethical sense check on data only studies that are put forward through UCLH (DT_09, DTC member, ST) - Determining if research is fit for purpose in respect of will it have benefits for the patients overall in respect of our population (DT_08, DTC member, ST), determining usefulness and feasibility of the of the work researchers are undertaking (DT_09, DTC member, ST) - Was envisaged as a group of patient and staff representatives who could review a project in detail to determine if ethically appropriate to proceed with it (DT_14, DTC attendee) - Essentially doing what an NHS rec or RA committee would do, like an ethical review to look at the appropriateness of a project and access to data (DT_12, DTC member, ST) - When checking for data ethics, for example, the data ethics around where is the data not necessarily physically, but what organizations have access to the data? because some of these projects are done in conjunction with private companies, so if there's a project with Microsoft, with GSK, we consider if there is a governing agreement around? What's going to happen with the data? Who owns the data? Who owns the IP of the project? and what we'll be done with that data. The good thing is that UCL should put these agreements together with these organizations, and there is normally a contract addendum for each project (DT_05, DTC member, ST). - I think my role is to focus on the ethical issues that particularly engage patients, and we know that those are, of course, confidentiality, the research using patient data should be for a health benefit and public benefit. There are issues around commercialization and intellectual property rights in which patients are concerned, because ultimately it's their data and they want to make sure that if that if it is used commercially that the benefits goes to you (DT_02, DTC member, PT).   Improve research application process   - Re outcome of DTC: the applications will improve, they'll be clearer, and the critical elements will be more obvious to DTC, and applicants will know what they are expected to provide (DT_09, DTC member, ST) - Provide input to a more streamlined process for approving data access applications (DTC_16, DTC member, PT; DT_12, DTC member, ST) with special reference to benefits for patients and the public (DTC_16, DTC member, PT) - UCLH as a research hospital is looking at the value of accessing real-world data, DTC streamlines access to real-world data (DT_12, DTC member, DT) - Get study requests for data- only studies through quickly so they don’t have to go through many different bodies (DT_04, DTC attendee), “streamlined approach” (DT_08, DTC member, ST) “more efficient” (DT_15, DTC attendee; DT_12, DTC member, ST), process to make anonymous data more streamlined at UCLH (DT_14, DTC attendee) - Approve research applications after they’ve gone through the Data Access Committee (DT_06, DTC member, PT) - Outcome should be that more people will put through research studies, or maybe people who are already doing research will use this process to get that approval process done faster. (DT_14, DTC attendee) - One of the goals that we want to achieve at the DTC is to also kind of delight the researchers because we want researchers to be happy and to be clear about how they carry out their research. - All the applications that we've gone through, I would say most of them have been with fairly mature researchers, but a smaller percentage, maybe 15% of the applications have come from fairly new researchers. They actually needed or they have needed our guidance to navigate how they are re going to achieve their research project. One example, there was a project that came through 2 months ago, and the researcher was a PhD student, and she was new to it and we actually arranged for a call with her afterwards, just with the committee, another staff committee member and myself, and we actually just coached her through how she should do things because she did not have that information at hand (DT_05, DTC member, ST). |
| Perception of current functioning of the DTC | - Is functioning as it should, have had a few applications where they have had an overview of how the research is presented and it's been quite helpful having the researcher there to present their application (DT_08, DTC member, ST). - Yes, seems to be doing what it’s expected to do. Challenging and giving pause for thought for ethical concerns (DTC_15, DTC attendee) - Yes, the staff and patient representatives are all very different, very diverse, all bring different ideas to the table (DT_14, DTC attendee)   Relating to new-ness of committee   - Sometimes it works quickly, sometimes it doesn’t work so quickly. The DTC is relatively new, they are still learning. Both the committee and those putting forward studies are learning the best way to do it and streamline the system. (DT_04, DTC attendee), Will get better as time goes on, it's only been a year (DT_09, DTC member, ST). - DTC still taking its shape. Things are still being ironed out in terms of information they receive and how the committee members can be a part of the conversation on what the requests are. Feels that it is still findings its general purpose. (DT_03, DTC member, ST), still process that's in evolution (DT_09, DTC member, ST) - Taking some time to bed down, and unclear how the RDAC feels it fits into this process. Might require more transparency about RDAC and DTC and how the two relate (DTC_16, DTC member, PT) - Yes, has taken away some red tape for the researchers and the individual researchers get feedback which is quite constructive. However it is only a year old so although it is functioning as it should, some improvements could be made (DT_12, DTC member, ST) |
| Description of DTC member and attendee’s role | Role of DTC members   - Review applications, make sure patient confidentiality is considered, make sure information is plausible and will benefit patients, and ultimately to decide whether the application goes ahead, needs amendments or to be resubmitted (DT_04, DTC attendee) - At the beginning their role wasn’t clearly defined, role has been moulded through the process. They created TOR together at the beginning. They weren’t sure whether it was like an ethics approval or clinical benefits of a successful trial etc. They realised that different steps of the process are already looking at these things, so by the time it reaches them it’s already had an ethical approval and clinical viability has been reviewed. The DTC provide a more generalist view that gives patients and general staff members a voice. To ensure ethical and governance arrangements are detailed in the application, and put ethics at the centre of what they are reviewing, but they are not the ethical approval (DT_03, DTC member, ST) - No operations or organisations of meetings, only review applications (DT_09, DTC member, ST) - ethics and feasibility, conflicts of interest with regards to the trust and how it’s funded. Positive scrutiny, challenging clarity & how to improve application (DT_08, DTC member, ST). - Members are there to fulfil the terms of reference and responsibilities of the DTC. Need to have gone through the relevant materials before meetings and take the role seriously (DTC_15, DTC attendee) - Role is to look at the potential benefits for patients and the public, looking at implications of research for intellectual property, commercialisation, and monetary benefit (DTC_16, DTC member, PT) - Based on presentations and applications they discuss, ask questions and respond to the application (DT_06, DTC member, PT) - To bring their experience to projects to assess if they are ethical (DT_14, DTC attendee) - Diverse group to bring their own lived experience and expertise to application process (DT_12, DTC member, ST) - A champion to safeguard the release of identifiable information, and help researchers identify what is lacking in their application (DT_12, DTC, ST). - Chair gives autonomy to the committee members. Like to leave decisions to the members, and then work on majority consensus. Chair role is to lead the DTC to ensure that every committee member has a face in in all decisions made, but also in discussions (DT_05, DTC member, ST). - Chair has got a lot of experience with data and technology, but also got that experience from the private sector as well as the public sector, so to lends experience in other spheres to the details when it comes to decision making and discussions and to help kind of demonstrate some of the benefits that the DTC (DT_05, DTC member, ST).   Other   - Project co-ordinator for DTC has an admin role, responds to researchers’ admin-related queries (not technical), sends out papers to DTC members, make sure there is an agenda, invite investigators, make sure they can attend the meetings, meeting notes, keep track of major points of discussion, shares the decision with researchers, fills in data concierge role when he isn’t available (DT_04, DTC attendee) - The research data access committee performs the feasibility review and advises the DTC (DTC_15, DTC attendee) - Data concierge is a single point of contact for researchers to help them understand what's going on during the development of the study, as before, the researchers would often be confused. Helps researchers to get access to data through stipulated pathway. Data concierge is person who is most familiar with forms and processes, who helps researchers fill them in correctly and then brings those forms to committee where discuss the technical aspects of its feasibility, information governance and so on. For DTC, doesn’t have a formal role, but makes sense to bridge the gap before getting to DTC (DT_14, DTC attendee) |
| Preparedness for role as DTC member (expectations, reality etc.) | - Less discussion between members than participant expected: Committee was set up immediately after covid, so has all been remote until very recently. Initially had expected that more information would be shared between them and there would be more discussions, but because of the format of the meetings, they answer researchers' questions and so mainly hear other’s views through their answers. Now that meetings will be held more in person, he thinks there will be more interchange of conversation between the members (DT_03, DTC member, ST) - Didn’t feel prepared in the sense that participant didn’t know what to expect, but it seemed that no one did (DT_03, DTC member, ST; DT_06, DTC member, PT). They went along with it and the role was moulded throughout the process. They created the TOR in their first couple meetings (DT_03, DTC member, ST). As much as someone could be prepared for a new role (DT_09, DTC member, ST) - Requires more time than expected (DT_09, DTC member, ST) - Those with an understanding of ethics (having done their own research) are potentially prepared better than some of the lay members. (DT_09, DTC member, ST) - Have research background and a keen interest in research which contributes to their knowledge and understanding needed for role (DT_08, DTC member, ST). - having an open mind is valuable and knowing how to work collaboratively with other groups helped with the role (DT_08, DTC member, ST). - Members of the DTC ask more questions than expected, and want to be clear on all the details. Some don’t understand the ins and outs of GDPR, for example, so need detailed explanations. No pushback, but healthy questioning (DTC_15, DTC attendee) - Didn’t have particular expectations other than people taking it seriously and listening to each other’s views, which they have done. Could have been given more of an outline of the role and an example of an application to understand better what was expected (DTC_16, DTC member, PT) - Data concierge role didn’t exist before, is new. They are figuring out problems as they go along. There is a bit of vagueness around that, but certainly nothing has been totally unexpected or untoward (DT_14, DTC attendee) - I really was excited when I first heard about the role. I was genuinely very excited, because there was 2 things that made me really want the role. It was my chance to genuinely give back and make a difference to the lives of people of Londoners, and hopefully, not just London. The intention is that we can influence other hospitals and other areas and other demographics internationally (DT_05, DTC member, ST). |
| Experience with the DTC so far | Positive   - Really hopes it continues, has been good and worthwhile committee (DT_04, DTC attendee), (DT_09, DTC member, ST). - Has found it very interesting and informative so far. Loves hearing all the novel things being trialled and hearing the perspectives of the patient members. Has learnt a lot (DT_03, DTC member, ST) - Interesting collaboration, looking at how you communicate with each other - technology not always the easiest for comms. Exposes us to different perspectives, using evidence- based research (DT_08, DTC member, ST). - Members take the time to understand the materials, are polite and courteous, and welcoming of other professionals and expertise. Good to hear their opinions. They’re always accepting of the advice that is given (DTC_15, DTC attendee) - Enjoyable so far, nice to contribute to the discussion by producing standardised questions for data requests to help applicants. (DTC_16, DTC member, PT) - Finds it fascinating (DT_06, DTC member, PT) - Made to feel welcome, at home and that the questions they ask are good questions (DT_06, DTC member, PT) - Been a positive experience (DT_14, DTC attendee) - A friendly, collaborative process with the researchers (DT_12, DTC member, ST).   Negative   - Some lack of clarity around potential commercialisation of data (DTC_16, DTC member, PT) |
| How JRO were made aware of DTC and their role |  |
| Perception of committee with staff and patient members (Including relationship between DTC staff and patient members) | - Hasn’t noticed any tensions, which is a good sign. Couldn’t tell who is staff and who is patient. No sense of division or conflict (DTC_15, DTC attendee) - Everyone is happy to speak up, although hybrid meetings aren’t the best for meeting people (DTC_16, DTC member, PT) - Members take it very seriously. people who have been recruited, really want to help with the research. They are curious, bring different questions, want to know detail about project and know why it will help (DT_14, DTC attendee) - “a healthy mix” (DT_12, DTC members, ST). - It's a very good point. We recognise right at the beginning that having a mix of lay members and staff members with different levels of maturity when it comes to the understand technology and medical terminology and techniques (DT_05, DTC member, ST).   Positive to have patient members   - Interesting for interviewee who is a clinician to have patient members on the panel and hear their take on things (DT_03, DTC member, ST) - Found it valuable working with patient representatives and the different stakeholders and knowing their concerns re safeguarding (DT_08, DTC member, ST). - Lay members have all participated in work similar to committee work so it’s not completely new to them (DT_12, DTC member, ST) - Clinicians bring a more clinical perspective, whereas patient members have a different perspective which bring helpful insights (DT_12, DTC members, ST).   Diverse expertise   - Large, diverse group with means that people have different questions which is really good. Staff with medical knowledge and patients with patient- related knowledge (DTC_06, DTC member, PT) - Works well, good to have this mixture. Each staff member has their own specialism, and patient members all have different qualities and some really pay attention to the detail in the applications, read all the papers. Also helps as sometimes basic questions need to be asked (DT_04, DTC attendee) - No division between patient and staff members – everyone has something individual to bring, all have breadth of knowledge and experience. They respect each other and are basically engaged and interested in the process (DT_14, DTC attendee) - Clinicians help explain clinical terms (DT_12, DTC member, ST) |
| Perceived benefits of the DTC | - They are following Best Practice guideline for using patient data, as need patient consent and involvement, needs patients to have a say in what happens with their data and this committee allows the patient voice to be heard (DT_03, DTC member, ST) - There have been problems of intellectual property before in research and innovation- Things originating in the NHS and then private companies testing them and taking them as their own. This committee contributes to protecting intellectual property (DT_03, DTC member, ST) - Gives you a slightly faster Streamlined process for data only trials, because there's not a backlog we seem to receive, review and vote (DT_09, DTC member, ST), speedier process, streamlined, (DT_08, DTC member, ST), efficiency (DTC_15, DTC attendee) - Tech and medical advancements will be brought around quicker resulting in better outcomes for patients and wider community (DT_08, DTC member, ST). - Will provide better quality research, which benefits wider population (DT_08, DTC member, ST). - Staff members get a better idea of the different parts of the ethics approval process, which helps. Gives the true impression that public and professional involvement is taken seriously by the hospital (DTC_15, DTC attendee) - Helps to focus attention on what needs to be considered when asking for patient data and to clarify applications – although it’s unclear if clarity is the DTC’s role (DTC_16, DTC member, PT) - Will benefit patients directly and patients in the future (DT_06, DTC member) - Gives chance or members of the public to get involved with research (DT_06, DTC member, PT) - Provides an internal committee for ethics review, where the default will be that all projects will have patient involvement, makes researchers think about patient needs in design. Will have broader purpose of showing transparency outside of DTC (DT_14, DTC attendee) - Will have real-world evidence units (units which look at real world data), which hopefully in time will look at real world data in every aspect of clinical practise. You need to use real-world data in real-time, which NHS rec process wouldn’t enable as takes a long time- need something like the DTC or DAP-R to speed it up (DT_12, DTC member, ST) - Many things are labelled as service evaluation because there was not a process outside of the NHS rec (like when participant was a junior doctor) but this process means it doesn’t have to be labelled as service evaluation. The DAP-R and DTC (committee within trust) enables a process where you are provided oversight, an ethical review, feasibility assessment and advice (DT_12, DTC member, ST) - On the public side some of the benefits that the DTC have brought is more transparency around projects because the great thing about having patient members is that we get the view of the public. Public concern on data commercialisation, management, storage, encryption are situations where a viewpoint of the public is important (DT_05, DTC member, ST). - a benefit for researchers is what kind of what I mentioned earlier with regards to being able to help them facilitate the process and also for them to understand ethics more as well, because not everyone understands data ethics, but for them to at least understand the basics of what the public is expecting, as well as what UCL is expecting (DT_05, DTC member, ST). - For the organization itself. UCL, because there's now that extra scrutiny that the DTC adds. Before the DTC existed, there was no ethical kind of measurement or ethical kind of checks (DT_05, DTC member, ST). - Some of the other benefits are to others. We hope to be able to take this DTC model and say, look, this is the way to do it right (DT_05, DTC member, ST). |
| Potential Risks of the DTC | - There are multiple stages in the application process and there isn’t full visibility of what is happening in the other stages. This can make it unclear to see who is checking what. So long as the roles are clearly defined then the risk is low or insignificant (DT_03, DTC member, ST), potential for replication with other stages e.g. RDAC and as result could delay process (DT_08, DTC member, ST). - Can't see any obvious risk of patients coming to harm as a direct consequence of this but could be unconscious collusion between panellists as may start agreeing with the loudest voice, there's a sort of group think thing that could take over (DT_09, DTC member, ST) - The full protocol wasn't always available, and sometimes the application wasn’t the same as what was presented so in light of those potential gaps, some risk can creep in (DT_09, DTC member, ST) - Sustaining buy-in from UCLH so that it can continue to be funded (DT_08, DTC member, ST). - Data protection law is due to change, with the removal of classification of anonymous data, which could affect the DTC (DTC_15, DTC attendee) - You start to see the same people all the time and understand their personalities, which could lead to tensions over time (DTC_15, DTC attendee) - Agreements about commercialisation has been put into the hands of the chair and is important to consider (DTC_16, DTC member, PT) - The process could be open to abuse if the committee was disinterested or conversely, under pressure in some way or incentivized to approve things that that maybe the external bodies wouldn't have approved. Also if there was fighting within the DTC, some members may want their voices heard and turn down projects if they think that they don't meet the standards they expect. (DT_14, DTC attendee) - A potential risk is in the future as technology becomes more advanced. Risk would be the committee if they lack understanding of the technology, (for example those that use free text or natural language processing) as will have to think of the patient identifiable information (DT_12, DTC member, ST) At the moment researchers build their own data sets using excel sheets and putting in the data so there is no problem yet (DT_12, DTC member, ST) - One of the kind of risks is that we don't get enough applications through and it's a general issue at a high level organization as well. Obviously, we want to be getting as many applications through as is feasible (DT_05, DTC member, ST). - One of the other risks which is, which will always be a risk, is that some applications may take longer to be approved. There was one particular which was a partnership with a private company, which took us a while to approve. However, the delays weren't actually due to us. We tried to get them on the call, but they didn't want to get on the call to actually answer questions. There were various other issues that happened. We wanted to see mentions about the contract addendums for the project, we wanted to see the contract, and then we didn't get it (DT_05, DTC member, ST). |
| Perception of DTC impact on research and application process | - Has made it easier to access data for data-only research requests (DT_03, DTC member, ST) - Has given research requests more transparency. Where before certain things may have been overlooked by clinicians and researchers, members of the public are able to give their say that adds an additional layer (DT_03, DTC member, ST) - As the DTC becomes more well known, in respect of templates they use for applications, will be pushed more quickly. Receiving more application which are more robust (DT_08, DTC member, ST). - More accessible process and people are becoming more aware of it (DTC_15, DTC attendee) - Improves the quality of applications (DTC_16, DTC member, PT)   **How data is used**   - Not necessarily how the data is used, but rather the fact that the data is there in the first place influences the development of data-only research at UCL and UCH- but this predates the DTC (DT_03, DTC member, ST) - Very early to tell, but moving in the right direction, building a good network (DT_09, DTC member, ST) - More robust process around processing and storage of data, cemented more every time they’re questioned (DTC_15, DTC attendee) - When the researchers present their research, due to more scrutiny, they are changing the way in which they present their proposal in respect of the data e.g., anonymity – how would they deal with the issue of some x-rays having the patient's name on them, etc (DT_08, DTC member, ST). - If you're either familiar with the system, or if you have a data person, it's very easy to say either just give me everything or just give me XYZ. But because of all the stages during this process where questions are asked whether they are feasibility questions or governance questions, are starting to see some consistency in terms of which data items researchers really think they need (DT_14, DTC attendee) - Will enable more real-time access to real-world data (DT_12, DTC member, ST)   **Oversight procedures, guardianship of data**   - Has definitely changed this- Has added a layer of reasonability to the application and increased the quality off the process, as they are patients and lay clinicians reviewing the applications (non-researchers). A part that has been overlooked historically. Previous scandals about not sharing data with the correct information governance procedures, and GDPR coming in just before Covid has really shaken things up making access to data much more stringent and making people feel that it isn’t safe to share data. (DT_03, DTC member, ST) - Doesn’t know enough about guardianship generally to comment, but it seems like it's taken very seriously within the scope of the DTC (DT_09, DTC member, ST) - Thinks there is enhanced guardianship of anonymous data with this group (DTC_15, DTC attendee) - Understands to some extent why data has to be anonymized, but finds it odd and if it was her data wouldn’t mind that it wasn’t anonymized (DT_06, DTC member, PT) - Doesn't think the DTC has changed the oversight procedures (DT_06, DTC member, PT) - More interest in data, have had internal media advertisements in the trust, lots of positive feedback and support from senior management (DT_08, DTC member, ST). - all the people in those high-level meetings can now see a bit more clearly in terms of what data do we have and what data is easily translatable into aggregate data for a research projects and large datasets (DT_14, DTC attendee) - now have a new team dedicated to research, specifically data management. So they are engaged. Now have a new process for managing hospital data to transform it into datasets that are usable for research in a consistent way (DT_14, DTC attendee) - Was common in the past for minor projects, to slip through the cracks. Either no one even thought of contacting the IG team or the IG team was too busy to respond to every single request, but everything is coming through this now. By design it has to get to IG (DT_14, DTC attendee) - Places without data trust committees made some applications hard to classify as there wasn’t a structure, but this provides an oversight structure and application for data only studies (anything that wasn’t an audit was a service evaluation, or classified as research, but not an intervention- now they have a place for data only studies). Each trust will or should need it eventually (DT_12, DTC member, ST)   **Speed of review and approval**   - There are bottlenecks in a small number of requests, but this must be at another point in the application process. Some months only have one or 2 or even no requests (DT_03, DTC member, ST) - Depends on the study, if they are requesting data from the last 100 years from a million people, there will be more queries and it will be less straight forward, but if information governance is straightforward it will be quicker (DT_04, DTC attendee) - Should be slightly better, but just for that small cohort of data only trials - Hard to know without the figures, but will also become more efficient once the group is more settled and used to the process (DTC_15, DTC attendee) - Applications are reviewed in a timely fashion and aren’t delayed, although sometimes they need to be resubmitted for quality and detail (DTC_16, DTC member, PT) - There's much more scrutiny, but there's much more feedback so it's helping the researcher to come back with a specific plan rather than walking away as they've actually been given appropriate feedback that's succinct so that they can then go and focus on that particular element within their application. As a result, has speeded up the research element (DTC_08, DTC member, ST). - Everyone who’s been through process knows they can do this again. As times go on, will become more streamlined and encourage more to get involved (DT_14, DTC attendee) - Difficult to compare to before, suspect there has been a small difference and will get more pronounced as time goes on. Some of the things that may have delayed in past may delay in future e.g. data sharing agreements, but won’t slow it down. As tackle problems, then some things will become higher priority (DT_14, DTC attendee) - Yes it has, though a few more things should be done to streamline more (transparency between DAP-R and DTC) (DT_12, DTC member, ST) - The DTC nature being another step will inevitably increase the amount of time it takes to process projects (DT_05, DTC member, ST).   Speed depends on the researchers:   - You have to dot all the Is and cross all the Ts on the application, and if this is done then it will go to the DTC quickly (DT_04, DTC attendee) - Availability of investigators: If the investigator is able to attend the DTC meeting and the queries answered then and there then it’s quick, but doesn’t always happen this way (DT_04, DTC attendee) - Responsiveness of researchers: DTC members do their bit, but also depends on how responsive and quick the researchers are to responding to the queries (DTC_04, DTC attendee) |
| Perceived impact on DTC members | - Overall a positive impact (DT_04, DTC attendee; DT_06, DTC member, PT) - Has had a positive impact on the participant as has increased participant’s interest in informatics, and seeing what is being done increases the participant’s interest in the possibility and potential for what can be done in the future (DT_03, DTC member, ST) - Been quite refreshing and enjoyable, (DT_06, DTC member, PT; DT_09, DTC member, ST) - But it takes a bit of time, and you have to take it seriously. Good it in that you see the breadth and complexity of work of some of the studies that you wouldn’t usually know about e.g. AI (DT_09, DTC member, ST); a pleasure to learn about researchers’ research (DT_06, DTC member, PT) - Sense of fulfilment for members, and can make a real difference and feel like they’re contributing. Makes them feel part of a research community. - Better idea of what goes into research (DT_06, DTC member, PT) in the NHS (DTC_15, DTC attendee); People are more aware of the intricacies of the research process (DTC_16, DTC member, PT; DT_12, DTC member, ST) and the challenges researchers face (DT_12, DTC member, ST) - Everyone has different expertise and perspectives, so people learn from each other (DTC_16, DTC member, PT) - looking at use of data more inclusively of patient public focus as well as staff members. And as a trust. Looking at it in a much more enriched detail. (DT_08, DTC member, ST) - Hopefully they quite enjoy being part of the DTC, particularly from having a sense of being more involved at an organizational level (DT_14, DTC attendee). - I think the benefits to all of the members, particularly the patient members. They've got a raised awareness of first of all our research works and what the purpose, what the researchers are trying to achieve (DT_05, DTC member, ST). |
| Perceived impact on researchers | - Would only be negative for a couple who don’t get their studies approved (DT_06, DTC member, PT), but overall a positive impact (DT_04, DTC attendee) - Anything that speeds up research applications is going to be good, nightmare going through HRA (DT_04, DTC attendee) - Feels that it has had a positive impact on researchers as they get feedback that helps them improve their requests, improve their considerations, (DT_03, DTC member, ST; DT_06, DTC member, PT), and maybe even makes them more aware of considering potential intellectual property and consider that these conversations need to take place at an earlier stage (DT_03, DTC member, ST) - Haven't had too much feedback from researchers yet but process is positive for those who get approval (DT_09, DTC member, ST) - Gives them a better idea of what goes into the process of approving and conducting research in the NHS (DTC_15, DTC attendee) - Has had a positive impact. Thinks it has been influential in respect of how people are looking at applications and proposals. The researcher walks away with comprehensive feedback so feel more comfortable knowing what the team is looking for and understanding how to make the amendment so that it does succeed in respect of approval. (DT_08, DTC member, ST) |
| Difficulties encountered | - Meeting monthly. Regular enough, but also not enough at times to make sure things keep moving (DT_04, DTC attendee) - DTC is new, so still ironing out the process and improving systems- this is currently happening (DT_04, DTC attendee) - Meeting on Teams has meant lack of discussions between the members, and having 4+ people on a teams meeting isn’t conducive to facilitating communication (DT_3, DTC member, ST) - Turnaround time between receiving applications (Wed) and the meeting (Mon). When have clinical obligations during the week then have to do DTC work on the weekend (DT_09, DTC member, ST) - quite few crossovers between RDAC and DTC (DT_08, DTC member, ST) - The whole process has had to be built from scratch, of which some of the attendee had done, not particularly by design, but just because it almost needed to happen. Had to create terms of reference for the committees and places to hold all of the data relevant to the projects and who can access those and who can amend those, etc. Had to also create all the document templates, meeting structures, etc which has been challenging (DT_14, DTC attendee) - The only difficulty is being around one specific project which I mentioned which was in partnership with a private company. The difficulties that we face there were being able to actually get them to give us the information we wanted, and to attend one of the meetings for a Q and A session, so we did have some difficulties there. These are communication kind of issues that can be resolved if we went through the right people (DT_05, DTC member, ST). - I think it was a communication issue, and also it was a slightly uncomfortable issue. When you get into these agreements, organization signs agreements with obviously lots of people or big corporations get involved with the NHS. That always gets people's attention, and provision made in those agreements for things like ethics, state ethics as well as data, security etc, and I think, yeah, you know the committee, the DTC wanted to see more detail. and we didn't get to see that detail (DT_05, DTC member, ST). |
| Suggestions to overcome difficulties/ improvements that can be made/ Recommendations | - Improve communication so that more researchers/people know about the DTC, get the word out to increase applications (DT_04, DTC attendee) - Meeting up with DTC from Birmingham to compare notes and learnings as they have been established for far longer now (DT_04, DTC attendee) - Would be good to do an evaluation later on to see how things have changed (DT_04, DTC attendee) - Meeting other members in person will help collaboration, and discussing matters in a more informal way with other members (DT_03, DTC member, ST) - Having more of an idea of what happens at other stages of the research application process, as knowing what other comments have been given before them helps them. They are currently working on this, would be good to get a summary sheet (DT_03, DTC member, ST) - getting the applications out a bit earlier so more time to review, providing panel with full protocol, having researchers meet online to help fill in any blanks early in process (DT_09, DTC member, ST) - Don’t always have the necessary expertise on committee, so need to go somewhere else to get that (DTC_16, DTC member, PT) - look at how the agenda is put together for the RDAC and whether we can streamline some of the processes because they look at the feasibility of the research, whereas we look at more of the ethical. Suggestion is to either for us to attend a couple of the RDAC sessions or to have access to some of the handover or the group discussions sheets in respect of that particular research. So that we know what questions and comments have been asked and to know you know how feasible the study is (DT_08, DTC member, ST; DT_12, DTC member, ST). - Transparency and visibility between R-DAC and DTC committee would help streamline the entire process (ie., R-DAC feedback needs to be visible to DTC members) (DT_12, DT member, ST) - Great to have loads of people involved who are invested, but sometimes it is helpful just to have a single person responsible for improving the infrastructure and the decision making, etc (DT_14, DTC attendee) - As more teams start looking at real world data, unsure whether one meeting a month will be enough. May need a larger DTC that meets in a cyclical sort of manner or a rota (DT_12, DTC member, ST) - I think things could improve is definitely around being able to show being able to demonstrate the value to the rest of the organization. I don't think we have a very good way of doing that at the moment at all. If we could have a quarterly, or maybe you know, even by year every 6 months if we could have a some sort of presentation to the management, but also to a wider group, and we've talked about doing that some more marketing. I think I think that would not only show other people the value you know, concrete in a concrete manner. But it will also benefit the organization hugely. because people will see that we're taking this seriously (DT_05, DTC member, ST).   Improvements in process   - Good that they receive detailed documents, but some are not all relevant and would be good to receive a more summarised document with more targeted answers to the questions they are answering. They are currently working on this- looking at the Dex form (DT_03, DTC member, ST) - As the committee is new and growing there are some adjustments to be made. Currently putting together a guide for the presenters for their presentations to cut it down (DT_06, DTC member, PT) - Are currently working on streamlining the process more (DT_04, DTC attendee). For example currently looking at providing guidance for people filling out the Dex form (request form researchers have to fill out) (DT_04, DTC attendee; DT_03, DTC member, ST) Reviewing which questions need to be asked on there (DT_03, DTC member, ST); Continuing to refine the requirements for the application, so that panel can get the key information up “front and centre” (DT_09, DTC member, ST) - Improving transparency between R-DAC and DTC (DT_12, DTC member, ST) |
| What works well | - Time taken for applications and good attendance at meetings. Good frequency of meetings too (DTC_15, DTC attendee) - Got set up and meetings are chaired appropriately (DTC_16, DTC member, PT) - Chairman very approachable (DT_06, DTC member, PT), Good relationship between chair and members of DTC, everyone can put forward their views, no frustrations (DT_14, DTC attendee) - Enough time given to read papers (DTC_16, DTC member, PT; DT_06, DTC member, PT) - Committee admin makes sure everything is organised (DT_06, DTC member, PT) - Flexibility of being in person and online for meetings (DT_09, DTC member, ST) Helpful to meet remotely online (DT_14, DTC attendee) - Rate at which decisions are made – 1 project a month approved (DT_14, DTC attendee) - Having Dexa system created already by software developers, with a much broader scope but by narrowing the scope down to just these projects, that's been good to have a single source of information that the researchers get to interact with directly (DT_14, DTC attendee) - The whole premise on which it was set up- having real people put in application for real data projects (DT_12, DTC member, ST) - Having a system by which to submit applications which is pretty streamlined – entry point and exit point work well (DT_12, DTC member, ST) |
| Contributing factors which help the DTC to work in this way/ work well | - Receiving feedback about the process from researchers is very useful (DT_04, DTC attendee) - The DTC members: many are volunteers, and they all give their 100%, attendance rate is good, they are dedicated members who care (e.g., one member couldn’t attend a meeting but met up with the project co-ordinator and the chair when he had just come out of the operating room to give his decision) (DT_04, DTC attendee); Data concierge and project co-ordinator meet on a weekly basis to touch base and keep things running (DT_04, DTC attendee) - Having investigator at the meeting really helps because some applications have very specific medical terms/ specialities they are referring to and this need explaining (DT_04, DTC attendee) - Process is very well planned, receiving documents in advance (DT_03, DTC member, ST); Paperwork comes out in the meetings are well organised and runs time, so those practical elements are quite good. There's always an agenda. There's a clear set of objectives. All the things that make all meetings go well. (DT_09, DTC member, ST). - Having a committee with members from very different backgrounds, with their common interest in informatics allowing them to speak the same language (DT_03, DTC member, ST) - Very collaborative and multidisciplinary (DT_03, DTC member, ST); collaborative and there is buy in from all the members (DT_09, DTC member, ST) The members have come together and gelled really well (DT_06, DTC member, PT), it's been very respectful because the collaboration has been really good (DT_09, DTC member, ST) - The data concierge guides researchers through the whole process and is the single point of contact (DTC_15, DTC attendee) - Project coordinator does all the important admin work to give DTC the gravity and formality it requires (DTC_15, DTC attendee), (DTC_16, DTC member, PT). - First and foremost, I think our members work very well. We've got a really good spread of skill sets of backgrounds, you know everything, you know, with the members. so we have a really good balance committee that here (DT_05, DTC member, ST). |
